# Mitigation of Acute Hepatotoxicity Induced by Cadmium Through Morin: Modulation of Oxidative and Pro-apoptotic Endoplasmic Reticulum Stress and Inflammatory Responses in Rats

**DOI:** 10.1007/s12011-024-04064-0

**Published:** 2024-01-19

**Authors:** Emin Sengul, Serkan Yildirim, İrfan Cinar, Samet Tekin, Yusuf Dag, Merve Bolat, Melahat Gok, Mohamad Warda

**Affiliations:** 1 Department of Physiology, Faculty of Veterinary Medicine, Atatürk University, Erzurum, Turkey; 2 Department of Pathology, Faculty of Veterinary Medicine, Atatürk University, Erzurum, Turkey; 3 Department of Pharmacology, Faculty of Medicine, Kastamonu University, Kastamonu, Turkey; 4 Department of Biochemistry, Faculty of Veterinary Medicine, Cairo University, Giza, Egypt

**Keywords:** Cadmium, Endoplasmic reticulum stress, Hepatotoxicity, Inflammation, Morin, Rat

## Abstract

Cadmium (Cd) is a toxic heavy metal with significant environmental health hazards. It enters the body through various routes with tissue accumulation. The relatively longer half-life with slow body clearance significantly results in hepatotoxicity during its liver detoxification. Therefore, researchers are exploring the potential use of herbal-derived phytocomponents to mitigate their toxicity. Here, we investigated, for the first time, the possible ameliorative effect of the phytochemical Morin (3,5,7,29,49-pentahydroxyflavone) against acute Cd-induced hepatotoxicity while resolving its underlying cellular mechanisms in a rat animal model. The study involved 50 adult male Sprague–Dawley rats weighing 200–250 g. The animals were divided into five equal groups: control, Cd, Morin100 + Cd, Morin200 + Cd, and Morin200. The 2nd, 3rd, and 4th groups were intraperitoneally treated with Cd (6.5 mg/kg), while the 3rd, 4th, and 5th groups were orally treated with Morin (100 and 200 mg/kg) for 5 consecutive days. On the 6th day, hepatic function (serum ALT, AST, ALP, LDH enzyme activities, and total bilirubin level) testing, transcriptome analysis, and immunohistochemistry were performed to elucidate the ameliorative effect of Morin on hepatotoxicity. In addition to restoring liver function and tissue injury, Morin alleviated Cd-induced hepatic oxidative/endoplasmic reticulum stress in a dose-dependent manner, as revealed by upregulating the expression of antioxidants (SOD, GSH, Gpx, CAT, and Nrf2) and decreasing the expression of ER stress markers. The expression of the proinflammatory mediators (TNF-α, IL-1-β, and IL-6) was also downregulated while improving the anti-inflammatory (IL-10 and IL-4) expression levels. Morin further slowed the apoptotic cascades by deregulating the expression of pro-apoptotic Bax and Caspase 12 markers concomitant with an increase in anti-apoptotic Blc2 mRNA expression. Furthermore, Morin restored Cd-induced tissue damage and markedly suppressed the cytoplasmic expression of JNK and p-PERK immunostained proteins. This study demonstrated the dose-dependent antioxidant hepatoprotective effect of Morin against acute hepatic Cd intoxication. This effect is likely linked with the modulation of upstream p-GRP78/PERK/ATF6 pro-apoptotic oxidative/ER stress and the downstream JNK/BAX/caspase 12 apoptotic signaling pathways.

## Introduction

Contamination of foods and animal feeds with heavy metals poses a severe global problem for human and animal health [1, 2]. Cadmium (Cd) is the seventh most toxic metal on a list of environmental pollutants [3, 4] and is widely used commercially in producing nickel-Cd batteries, coatings, plastic materials, toys, agricultural fertilizers and herbicides, thermometers, electronic devices, cosmetics, and dyes [5, 6]. It is extremely harmful to living organisms since it has been extensively detected in soil, food, air, and water [7]. It is abundant in nature, enabling its accumulation in the tissues of livestock grazed on contaminated pasture with possible milk contamination [8–10]. It accumulates mainly in the liver and kidneys, in addition to other organs [11–13]. Its renal and hepatic accumulation aggravates inflammatory and oxidative stress that ends with cellular apoptosis of the affected organs [14, 15]. The liver is seriously affected by Cd intoxication, manifested by altered biomarkers of hepatic function, with concomitant cellular degeneration and fatty infiltration that end with accelerated cellular necrosis [16]. In addition, studies have reported that Cd induces inflammation, oxidative damage, endoplasmic reticulum (ER) stress, and apoptosis in the liver [15–17].

Many compounds with protective antioxidant, anti-inflammatory, and anti-apoptotic effects were previously used against Cd-induced hepatic damage [18–20]. Flavonoids are compounds abundantly found in fruits and vegetables. Their strong antioxidant, anti-inflammatory, and anti-apoptotic effects have been thoroughly investigated. Morin (2′,3,4′,5,7-pentahydroxyflavone), a flavonoid, is abundantly found in many plants, such as members of the *Moraceae* family [21]. It has various biological activities, including antioxidant [22], anti-inflammatory, anti-apoptotic [23], and anticancer [24] properties. Despite the proven protective effects of Morin against Cd-induced neuropathy in rats [25], its potential hepatoprotective role in acute Cd hepatic intoxication has not been resolved. Therefore, this study aimed to elucidate this possible hepatoprotective effect of morin. The antioxidative/pro-apoptotic effects on ER stress, as well as the anti-inflammatory and anti-apoptotic effects of Morin, were also studied. Then, the cellular mechanism behind these effects was further resolved.

## Methods

### Chemicals and Reagents

Cadmium chloride (CdCl_2_; 99.99% purity), Morin hydrate (CAS number: 654055–01-3), and all other reagents were supplied by Sigma Chemical Co. (St. Louis, MO, USA). Rat ELISA kits were obtained from Sunred Biological Technology (Shanghai, China).

### Animals and Experimental Design

Male Sprague–Dawley rats (weighing 200–250 g, 10–12 weeks old) were purchased from the Medical Experimental Research and Application Center, Atatürk University (Erzurum, Turkey). The Atatürk University Ethical Committee approved the experimental protocol for our experiment (approval no: 2020/132).

The rats were housed under standard laboratory conditions (24 ± 1 °C, 45 ± 5% humidity, and 12-h light/dark cycle). Rats had access to a commercial pellet diet (Bayramoglu Feed and Flour Industry Trade A. C. Erzurum) and water ad libitum throughout the whole study. After 1 week of acclimatization, animals were grouped into five experimental groups (control, Cd, Morin100 + Cd, Morin200 + Cd, and Morin200) with ten rats each following the design below:

- Control: One milliliter of distilled water was orally given to each rat for 5 consecutive days.
- Cd: Rats were intraperitoneally injected (i.p.) with Cd (6.5 mg/kg) [26] for 5 consecutive days.
- Morin100 + Cd: Morin (100 mg/kg) was orally administered to each rat [22] followed by Cd (6.5 mg/kg, i.p.) injection after 1 h of Morin administration for 5 consecutive days.
- Morin200 + Cd: Morin (200 mg/kg) was orally administered to each rat [22] followed by Cd (6.5 mg/kg, i.p.) injection after 1 h of Morin administration for 5 consecutive days.
- Morin200: Morin (200 mg/kg) was orally administered to each rat for 5 consecutive days.

On the sixth day of the experiment, live animals were weighed and then euthanized by mild sevoflurane anesthesia, and intracardiac blood samples were collected. All rats were sacrificed immediately after blood sampling by decapitation, and abdominal laparotomy was performed to obtain the liver. After weighing, each liver was divided into two parts: the 1st was snap frozen in liquid nitrogen and stored at − 80 °C to be used for biochemical and gene expression studies, and the 2nd was immediately flushed with saline and then taken into 10% formaldehyde for histopathological and immunofluorescent examinations.

### Preparation and Analysis of Serum Samples

The serum samples were recovered from blood by centrifugation at 3000 rpm for 10 min. Serum activity of aspartate transferase (AST), alanine aminotransferase (ALT), alkaline phosphatase (ALP), lactate dehydrogenase (LDH), and total bilirubin (TB) levels were analyzed in an autoanalyzer at Atatürk University Veterinary Faculty Veterinary Diagnosis and Analysis Laboratories.

### Biochemical Analysis

Liver tissue homogenates for oxidative stress and inflammation biomarker analysis were obtained as previously described [27]. Oxidant and antioxidant parameters (MDA, SOD, GPx, GSH, Nrf2, and CAT), proinflammatory cytokines (TNF-α, IL-6, IL-1β, IFN-γ), and anti-inflammatory cytokines (IL-10, IL-4) in the recovered liver tissue supernatants were measured using rat ELISA kits according to the manufacturer’s instructions. The reading was performed at an absorbance of 450 nm by an ELISA plate reader (Bio-Tek, Winooski, VT, USA).

### Histopathological Examinations

Rat liver tissues were prepared for hematoxylin–eosin (HE) staining. Tissue samples were fixed in phosphate-buffered 10% formaldehyde solution for 48 h and embedded in paraffin wax blocks. Samples were cut into 4-µm sections for each block. Sections were then dewaxed and hematoxylin–eosin stained, followed by alcohol gradient dehydration prior to being examined under a light microscope (Olympus BX 51, Tokyo, Japan).

Sections were evaluated as absent (-), mild ( +), moderate (+ +), and severe (+ + +) according to their histopathological features.

### Immunofluorescence Examination

The 4-μm sections were taken on adhesive slides, deparaffinized, dehydrated, and washed with PBS. Endogenous peroxidase was inactivated in 3% H_2_O_2_ for 10 min. Then, the samples were boiled in 1% antigen retrieval (citrate buffer (pH + 6.1) 100X) solution and cooled to room temperature. Sections were incubated with protein block for 5 min to abolish nonspecific background staining. Then, the primary antibody (JNK cat no: sc-514539, dilution ratio: 1/100, US) was added according to the manufacturer’s instructions. This was followed by the immunofluorescence 2nd antibody marker (FITC cat no. ab6785, diluent ratio 1/500, UK) and dark incubation for 45 min. Then, the primary antibody (p-PERK cat no. sc-7383, dilution ratio 1/100, US) was dripped onto the sections and incubated according to the manufacturer’s instructions. A secondary immunofluorescence antibody was used as a secondary marker (Texas Red cat no. ab6719, diluent ratio 1/500, UK) and kept in the dark for 45 min. Then, DAPI with mounting medium (cat no. D1306, dilution ratio 1/200, UK) was dripped onto the preparations and kept in the dark for 5 min. Afterward, the stained sections were covered with a coverslip and examined under a fluorescence microscope (Zeiss AXIO, Germany).

### Gene Expression Analysis

GRP78, CHOP, ATAF6, p-IRE1, sXBP1, Bax, Bcl-2, and Caspase-12 mRNA expression levels in tissue samples were determined by the real-time PCR method and evaluated between groups as follows.

### RNA Extraction

The tissue samples were homogenized in Tissue Lyser II (Qiagen) in the presence of liquid nitrogen. Total RNA was then extracted (QIAcube Connect Qiagen kit for RNA isolation), and the recovery was estimated.

### Reverse Transcriptase Reaction and cDNA Synthesis

cDNA was reverse transcribed from total RNA using the high-capacity cDNA Reverse Transcription Kit (Applied Biosystems). Ten micrograms of RNA was used for each reaction, and reactions were performed in a thermal cycler (Veriti 96-Well Fast Thermal Cycler, Applied Biosystems) following the manufacturer’s protocol. The recovery of cDNA was then determined using nanodrop spectrophotometry (EPOCH Take3 Plate, BioTek) prior to storage at -20 °C [28].

### Real-Time Quantitative PCR

The quantification of each gene was performed on the StepOnePlus Real-Time PCR System (Applied Biosystems). Genes were quantified together with the ACTB (β-actin) gene as a housekeeping gene using the TaqMan Gene Expression kit (TaqMan™ Gene Expression Master Mix, Applied Biosystems) as described in our previous study (Cinar et al. 2021). One hundred nanograms of cDNA was used for each gene with 40 cycles run. Ct values were converted to delta Ct, and the obtained results were statistically evaluated by the SPSS 20.00 package software program.

### Statistical Analyses

Quantitative and semiquantitative values obtained at the end of the studies were evaluated using the Tukey test after one-way ANOVA, used in the statistical analysis of more than two independent groups in the SPSS 20.00 statistical data program.

The Kruskal‒Wallis test, a nonparametric test, was used to analyze the differences between the groups in the semiquantitative data obtained in the histopathological examination, and the Mann‒Whitney *U* test was used for the comparison of the paired groups.

To determine the intensity of positive staining from the pictures obtained as a result of the staining, five random areas were selected from each image and evaluated in the ZEISS Zen Imaging Software program. Data were statistically defined as the mean and standard deviation (mean ± SD) for % area. One-way ANOVA followed by the Tukey test was performed to compare the numbers of positive immunoreactive cells and the numbers of immunopositive stained areas obtained from the analysis with healthy controls. As a result of the tests, *p* < 0.05 was considered significant, and the data are presented as the mean ± SD.

## Results

### Effects of Morin and Cd on Body Weight and Liver Weights

Table 1 presents the effects of Morin and Cd on body weight and liver weights in rats. At the beginning of the experiments, the body weights of the rats did not differ between the experimental groups. However, at the end of the experimental study, there was a decrease in body weight in the Cd, Morin100 + Cd, and Morin200 + Cd groups compared to the control (*p* < 0.05). The body weight in the Morin200 group did not differ from that in the control group (*p* ˃ 0.05), while liver weights did not differ between the experimental groups (*p* ˃ 0.05, Table 1).

**Table 1:** Live weights and liver weights of the experimental group rats at the beginning and end of the experimental study

Parameters	Control	Cd	Morin100 + Cd	Morin200 + Cd	Morin200
Initial body weights (g)	217.00 ± 11.92^a^	220.25 ± 12.96^a^	218.13 ± 10.13^a^	213.62 ± 9.80^a^	212,5 ± 11.64^a^
Finished body weights (g)	236.63 ± 5.97^a^	203.88 ± 12.99^b^	216.13 ± 8.25^b^	223.63 ± 5.21^bc^	234.38 ± 9.74^ac^
Liver weights (g)	10.65 ± 1.02^a^	10.13 ± 0.85^a^	10.35 ± 0.8^a^	10.38 ± 0.71^a^	10.42 ± 1.78^a^

a-b, *p* < 0.01; a-bc, b-ac, *p* < 0.05

### Effects of Morin and Cd Administration on Serum Liver Enzyme Levels

Table 2 demonstrates that Cd caused significant increases in serum ALP, ALT, AST, and LDH levels compared with the control and Morin200 groups (*p* < 0.05). The low dose of Morin prevented the increase in Cd-induced enzyme levels, but not significantly (*p* > 0.05). The high dose of Morin significantly decreased the Cd-induced increase in ALP and ALT enzyme levels (*p* < 0.05).

**Table 2:** Effects of Morin and Cd treatment on liver enzyme levels

Groups	ALP (U/L)	ALT (U/L)	AST (U/L)	TB (mg/dL)	LDH (U/L)
Control	91.75 ± 19.61^a^	43.7 ± 3.08^a^	115.02 ± 13.68^a^	0.05 ± 0.01^a^	527.75 ± 70.29^a^
Cd	181.75 ± 27.6^b^	92.80 ± 8.64^b^	174.55 ± 25.52^b^	0.07 ± 0.01^a^	700.25 ± 65.93^b^
Morin100 + Cd	128.50 ± 28.26^bc^	79.35 ± 8.61^bc^	157.73 ± 30.45^ab^	0.06 ± 0.01^a^	680.50 ± 84.33^ab^
Morin200 + Cd	113.60 ± 29.5^ac^	68.43 ± 10.68^ac^	133.06 ± 17.77^ab^	0.06 ± 0.01^a^	628.00 ± 96.68^ab^
Morin200	97.25 ± 16.46^a^	44.28 ± 5.30^a^	120.63 ± 13.14^a^	0.06 ± 0.01^a^	542.75 ± 60.09^a^

a-b, *p* < 0.01; a-bc, b-ac, *p* < 0.05

### Effects of Morin on Cd-Induced Hepatic Oxidative Stress

**Fig. 1:**
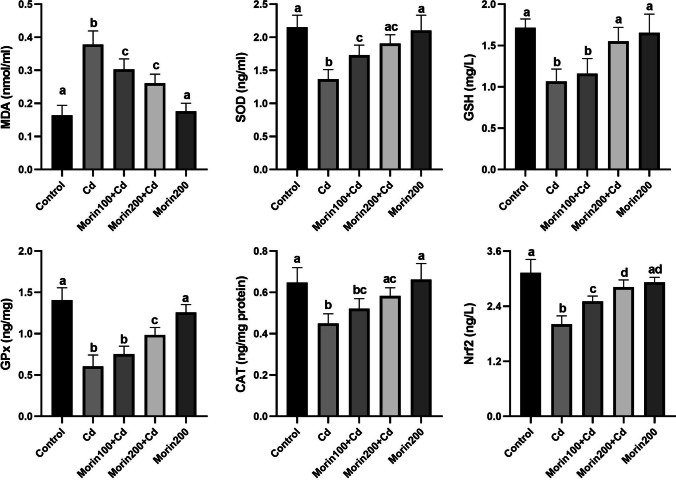
Effects of Morin against Cd-induced hepatic oxidative stress in rats. Different letters (a–d) on the columns show a significant difference (*p* < 0.05). Values are expressed as mean ± SEM

The effects of Morin administration on Cd-induced hepatic oxidative stress are summarized in Fig. 1. The MDA level in the Cd group was significantly higher than that in the control group (*p* < 0.05). Both doses of Morin significantly (*p* < 0.05) corrected the observed increase in Cd-induced lipid peroxidation, while treatment with Morin alone did not cause a change (*p* > 0.05) in the MDA level. The antioxidant GSH and Nrf2 levels and the activity of SOD, GPx, and CAT enzymes were significantly decreased in the Cd group compared to the control, Morin200 + Cd, and Morin200 groups (*p* < 0.05). The low dose of Morin prevented the observed Cd-induced decrease in SOD activity and Nrf2 levels (*p* < 0.05), with no significant effect on the decrease in GSH levels or GPx and CAT activities.

### Effects of Morin on Cd-Induced Hepatic Inflammation

**Fig. 2:**
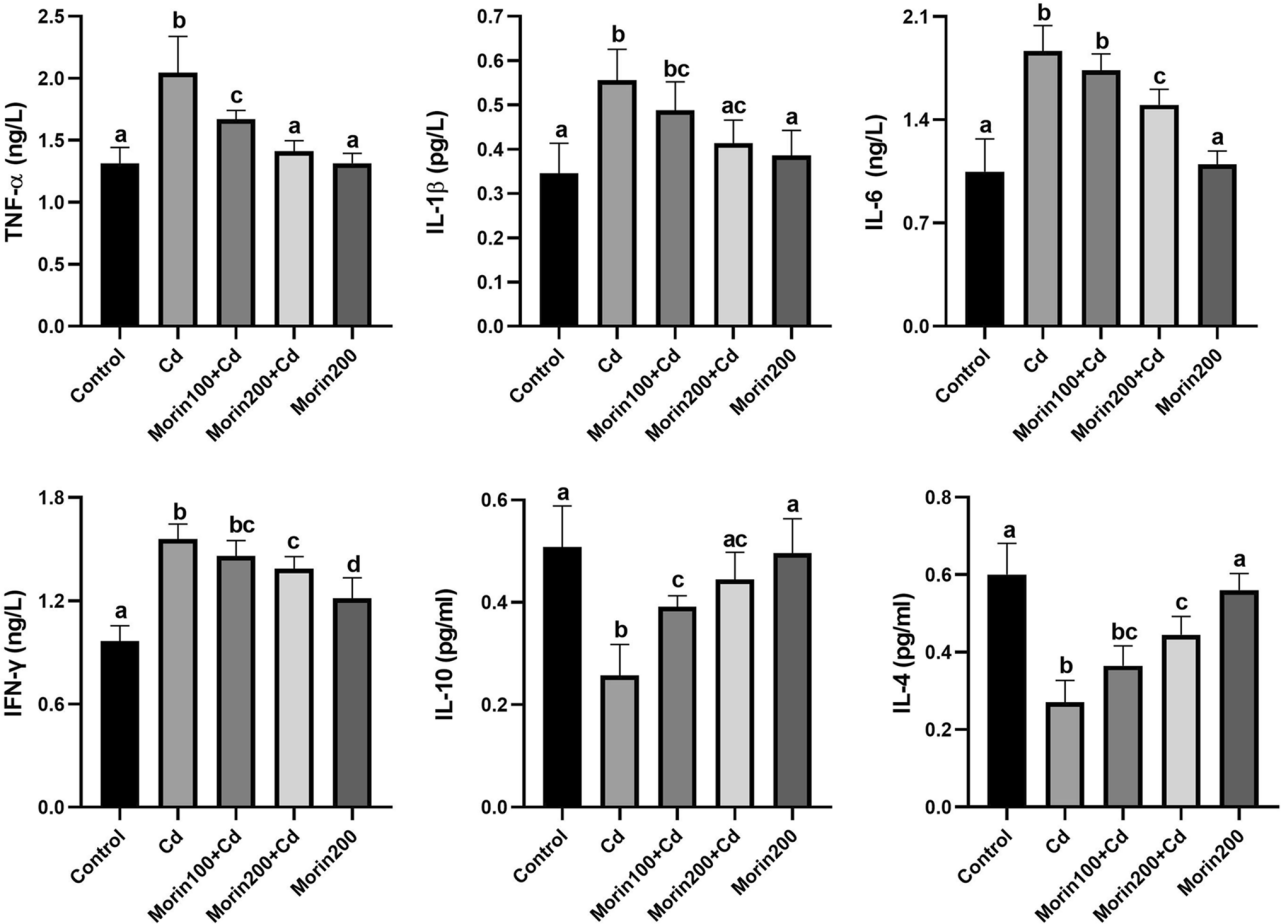
The effects of Morin against Cd-induced hepatic inflammation in rats. Different letters (a–d) on the columns show a significant difference (*p* < 0.05). Values are expressed as mean ± SEM

The effects of Morin administration on Cd-induced hepatic inflammation are summarized in Fig. 2. TNF-α, IL-1-β, and IL-6 levels were significantly increased in the Cd group compared to the control, Morin200 + Cd, and Morin200 groups (*p* < 0.05). The low dose of Morin decreased the increase in TNF-α, IL-1-β, IL-6, and IFN-γ levels, but the decrease in IL-1-β, IL-6, and IFN-γ levels was not significant (*p* > 0.05). IL-10 and IL-4 levels were significantly decreased in the Cd group compared to the control, Morin200 + Cd, and Morin200 groups (*p* < 0.05). While the low dose of Morin prevented the decrease in IL-10 levels, it could not significantly prevent the decrease in IL-4.

### Effects of Morin on Cd-Induced Hepatic ER Stress

**Fig. 3:**
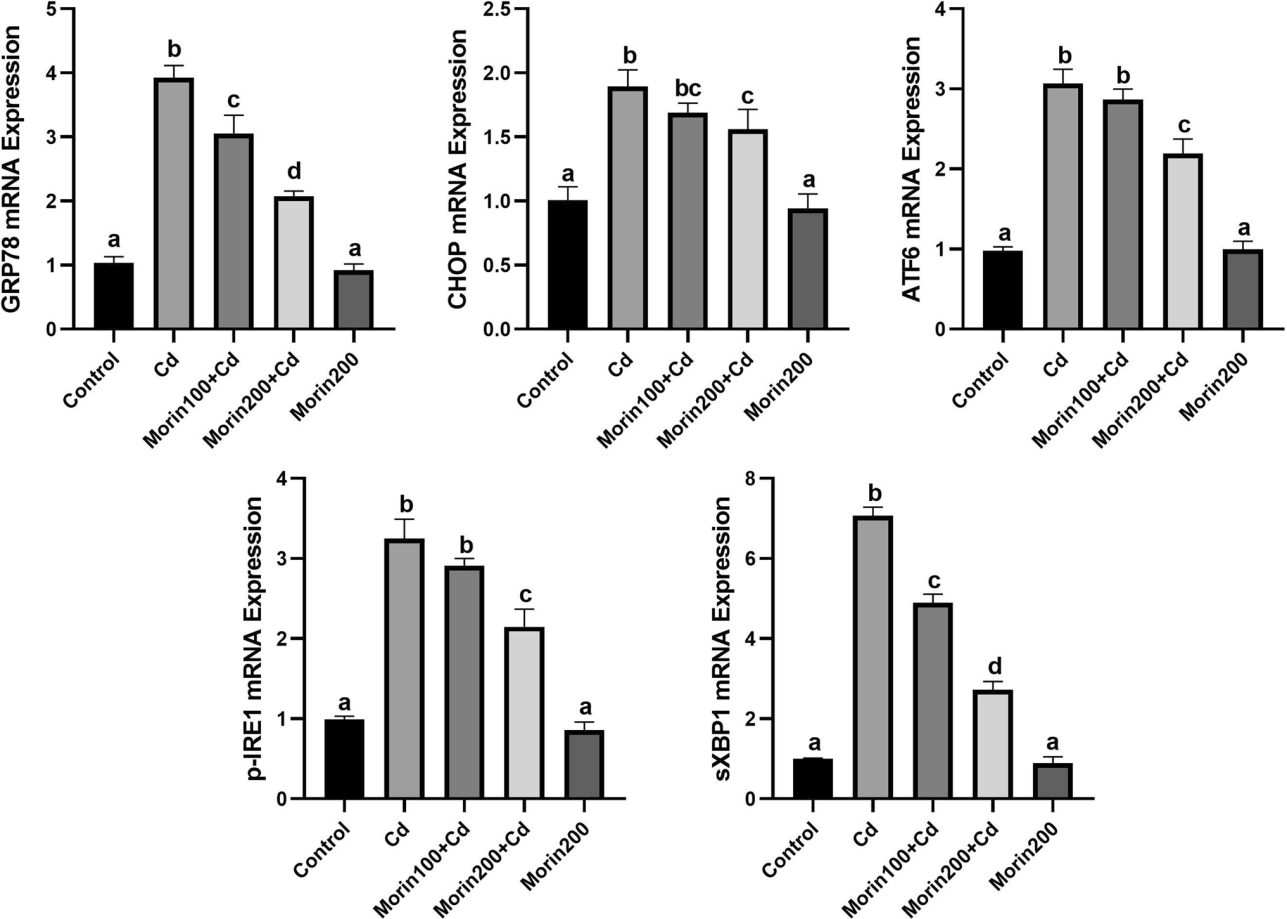
The effects of morin against Cd-induced hepatic ER stress in rats. Different letters (a–d) on the columns show a significant difference (*p* < 0.05). Values are expressed as mean ± SEM

The effects of Morin administration on Cd-induced hepatic ER stress are summarized in Fig. 3. Significant increases in the mRNA expression levels of GRP78, CHOP, ATF6, p-IRE1, and sXBP1 were observed in the Cd group compared to the other groups (*p* < 0.05). The low dose of Morin significantly prohibited this increase in GRP78 and sXBP1 mRNA expression levels (*p* < 0.05).

### Effects of Morin on Cd-Induced Apoptosis

**Fig. 4:**
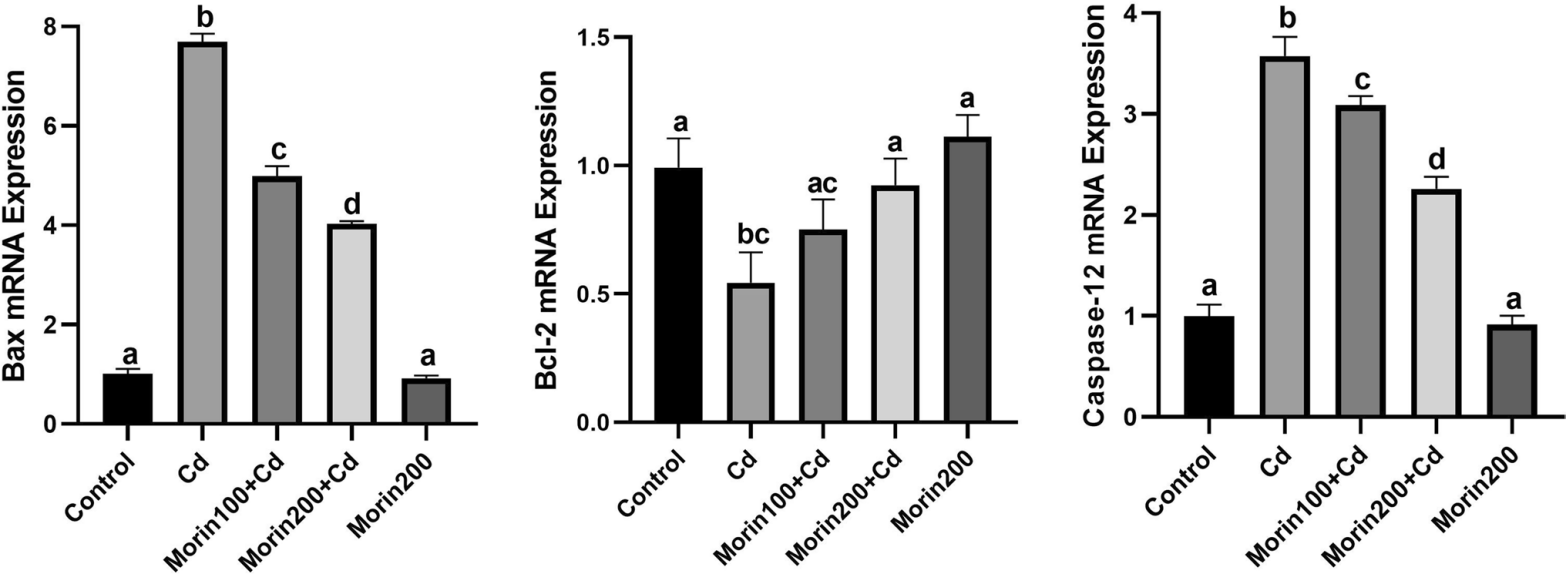
The effects of Morin against Cd-induced hepatic ER stress in rats. Different letters (a–d) on the columns show a significant difference (*p* < 0.05). Values are expressed as mean ± SEM

The effects of Morin administration on Cd-induced apoptosis are summarized in Fig. 4. Bax and Caspase-12 mRNA expression levels were increased in the Cd, Morin100 + Cd, and Morin200 + Cd groups compared to the control and Morin200 groups (*p* < 0.05). The Bcl-2 mRNA expression level decreased significantly in the Cd group compared to the other groups. Morin, however, restrained the increase in Bax and Caspase-12 mRNA expression levels in a dose-dependent manner (*p* < 0.05).

### Histopathological Findings

Histopathological findings are shown in Table 3. The normal standard hepatic architecture observed in hepatic tissues in the control and Morin200 groups was greatly altered with severe observed damage in the Cd group, manifested with significant acinar degeneration and necrosis and marked sinusoidal and parenchymal vessel hyperemia. The Morin100 + Cd group, however, recorded moderate hepatocyte degeneration with limited coagulative necrosis and vascular hyperemia in the acinar region. Mild vacuolar degeneration and vascular hyperemia were observed in the acinar region in the Morin200 + Cd group with significant hepatic recovery (*p* < 0.05) when compared with the Cd group.

**Table 3:** Scoring of histopathological findings in liver tissues of rats in experimental groups

Groups	Degeneration in hepatocytes	Necrosis in hepatocytes	Hyperemia in the veins
Control	-	-	-
Cd	+ + +	+ + +	+ + +
Morin100 + Cd	+ +	+	+ +
Morin200 + Cd	+	-	+
Morin200	-	-	-

### Immunofluorescence Findings

**Fig. 5:**
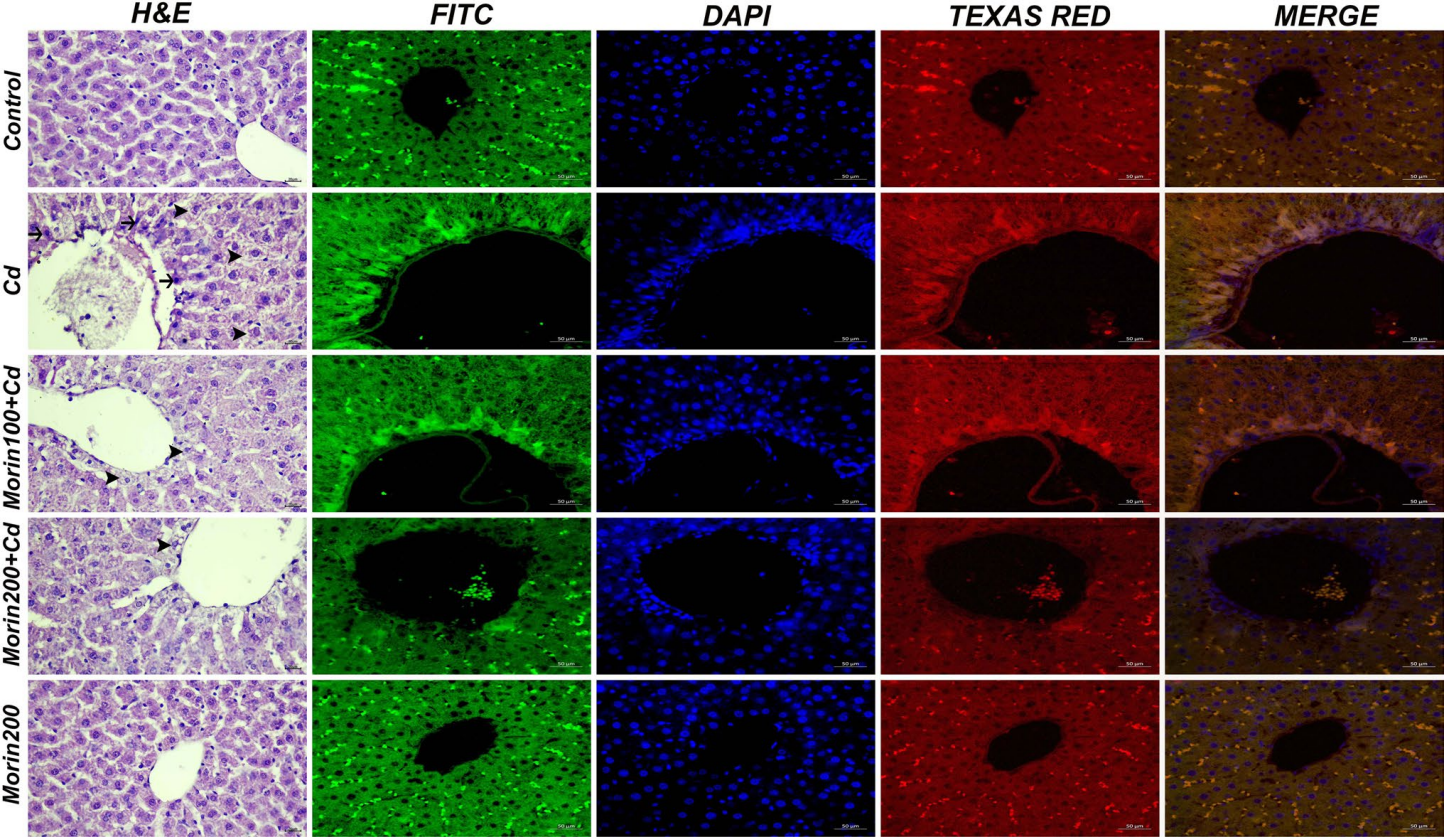
Liver tissue, hepatocyte degeneration (arrowheads), necrosis (arrows), hyperemia, H&E, bar 20 µm, JNK expression (FITC), p-PERK expression (Texas Red), IF, bar 50 µm

Immunofluorescent findings are summarized in Table 4. For tissue samples from the control and Morin200 groups, the immunofluorescence expression of JNK and p-PERK was evaluated as negative. The Cd group, however, demonstrated a severe increase in the cytoplasmic expression of both JNK and p-PERK proteins in the acinar region. This observed increase was moderately reduced in the Morin100 + Cd group but significantly abolished in the Morin200 + Cd group compared with the Cd group Fig. 5.

**Table 4:** Statistical analysis results of immunofluorescence findings in liver tissues of rats in experimental groups

Groups	JNK expression	p-PERK expression
Control	21.41 ± 3.25^a^	20.66 ± 4.72^a^
Cd	80.68 ± 5.85^b^	81.26 ± 5.96^b^
Morin 100 + Cd	59.45 ± 3.87^c^	60.57 ± 5.75^c^
Morin 200 + Cd	41.63 ± 5.41^d^	39.94 ± 5.61^d^
Morin 200	20.56 ± 5.39^a^	20.8.9 ± 4.87^a^

a, b, c, d: Different letters in the same column represent a statistically significant difference (*p* < 0.05)

## Discussion

Cd is a toxic heavy metal with a recorded health hazard [29]. It has wide source contamination, e.g., PVC products with color pigments and Ni–Cd batteries [30]. In contaminated areas, it has a great tissue penetrability even through house dust [31]. However, the respiratory tract is the major route for entry, and Cd can also infiltrate through the digestive tract. Following its circulation access, it accumulates in the liver and kidney, inducing hepatorenal toxicity [29]. Therefore, attempts to minimize Cd-induced damage have been seriously investigated. Morin, on the other hand, is a flavone phytochemical with reported anti-inflammatory, antioxidant, and anti-apoptotic effects [22, 23, 32]. To our knowledge, this is the first study to resolve the hepatoprotective effect of Morin against acute Cd intoxication with accompanying oxidative stress, inflammation, and ER stress–mediated apoptosis. Therefore, we aimed to evaluate the possible hepatoprotective effect of Morin on Cd-induced liver toxicity in rats.

Serum hepatic biomarkers, including AST, ALT, ALP, LDH enzymes, and bilirubin, reportedly increase in hepatic injury [33], including Cd-induced hepatotoxicity [34]. Similarly, our results demonstrated a significant increase in these biomarkers in Cd-intoxicated livers. Rizvi et al. [35] found that Morin treatment reduced this increase in serum hepatic biomarkers during acetaminophen-induced liver damage. Consistent with this finding, Morin treatment significantly lessened this increase in serum hepatic biomarkers after Cd intoxication and incredibly abolished this increase at high doses.

Oxidative stress leads to cellular damage to many biological molecules, such as DNA, proteins, and lipids. This damage can occur in cells by reactive oxygen species (ROS) [36, 37]. ROS cause lipid peroxidation of cellular membranes, and membrane integrity is impaired. To prevent these effects of oxidative stress, there are several antioxidant defense mechanisms in the cell. SOD, CAT, GPx, and GSH are essential in this defense mechanism (Ighodaro et al. 2018). The intracellular oxidant and antioxidant balance systems are disrupted when Cd accumulates in cells. The elimination and production of ROS are controlled under redox equilibrium. The cell’s antioxidant defense system achieves redox homeostasis. SOD, CAT, GSH, GPx, and Nrf2 are intracellular antioxidants that try to control the redox balance of the cells. Increasing ROS aggregates cellular oxidative and leads to the breakdown of intracellular lipids. The developed lipid peroxidation due to the lipid breakdown is coherent with the increased level of cellular MDA [38]. Studies conducted in experimental animals have reported that Cd causes oxidative stress and cell damage [39],Kunle et al. 2017). Fang et al. [40] found that SOD, CAT, and GPx activities and GSH and Nrf2 levels decreased, while MDA levels increased in Cd-induced hepatotoxicity. Another study revealed that SOD, CAT, and GPx activities and GSH levels decreased in liver damage as a result of Cd administration [41]. Our study determined that Cd administration stimulated oxidative stress in the liver. MDA levels increased due to lipid peroxidation, while GSH, and Nrf2 levels, and SOD, CAT, and GPx activities decreased. Conversely, Morin has a proven protective effect against toxicity induced by many agents [42, 43]. Consistently, our finding demonstrated that Morin administration significantly ameliorated Cd-induced oxidative stress in rats.

Cellular inflammatory response, on the other hand, is associated with chronic diseases, especially cancer, diabetes, and cardiovascular and neurological diseases. However, it has been reported that various transcription factors, such as AP-1, NFĸB, and p53, are activated under oxidative stress [44]. Cd induces inflammation by increasing proinflammatory cytokine levels and decreasing anti-inflammatory cytokine levels [45, 46]. Additionally, it has been determined that Morin prevents/reduces experimentally induced inflammation [23, 47]. Our findings confirm the previous finding that Cd-induced hepatic inflammation increased proinflammatory cytokine levels and decreased anti-inflammatory cytokine levels. Here, we demonstrated the protective anti-inflammatory role of Morin against Cd-induced hepatic inflammation.

Oxidative damage and ER stress are interrelated in the pathogenesis of various diseases. The ER is a membrane-bound organelle with critical physiological processes such as calcium storage, lipid production, protein folding, and biosynthesis [48, 49]. The ER is, therefore, a highly vulnerable organelle to intracellular and extracellular stimuli. Any cellular oxidative induces ER stress with inhibition of protein folding capacity of stressed ER. This consequently results in accumulation of misfolded proteins [50]. In a normal cell, PERK, ATF6, and IRE1α are tightly bound to GRP78. Any cellular event that induces ER stress has to increase the accumulation of cellular unfolded or misfolded protein. Accordingly, these three transmembrane proteins are separated from GRP78 to combine with the unfolded proteins. Subsequently, p-PERK and IRE1α are activated by trans-autophosphorylation and ATF6 proteolytic processing [51]. Experimentally, hepatic ER stress can be evoked by Cd in rats [16], with parallel increase in expression levels of the member of upstream signaling pathway including GRP78, CHOP, ATF6, p-IRE1, sXBP1, and p-PERK. Pandey et al. [52] determined that Morin attenuates ER stress in diabetic hepatotoxicity in rats. Another study reported that Morin has an ER stress-reducing effect in methotrexate-induced testicular toxicity in rats [53]. Consistent with the previous results, our study revealed that Cd-induced ER stress with significant mRNA over-expression of GRP78, CHOP, ATF6, p-IRE1, sXBP1, and p-PERK genes compared with the control group. Morin, however, abrogated the upstream signaling pathway of Cd-induced hepatic ER stress.

Moreover, the ER-induced apoptosis is mediated by caspase pathway activation. This pathway is independent of mitochondrial and death receptors but possibly mediated by caspase-12. Caspase-12 is activated by ER stress and then translocates from the ER to the cytosol, where it activates the effector caspases such as caspase-3 [54]. A study reported that Cd treatment causes an increase in neuronal caspase-12 expression levels [55]. Rizvi et al. [56] revealed that Morin prevents the increase in caspase-12 induced by tert-butyl hydroperoxide in primary hepatocytes. Parallel with this finding, our investigation demonstrated that Cd significantly increased caspase-12 expression in liver tissue and Morin administration dose-dependently prevented this increase.

As a result of experimental studies, it has been determined that ROS have a crucial role in inducing the apoptotic pathway under pathological and physiological conditions [57]. Apoptotic cell death pathways are mitochondrial pathways induced by members of the Bcl-2 protein family, which includes Beclin-1, Bax, and Bcl-2 [58]. The imbalance in the Bax/Bcl-2 ratio, which may occur due to various stimuli, causes an increase in cytochrome c levels in the cytoplasm, activating caspase enzymes stimulated by direct or indirect ROS activity, and apoptosis develops [59]. Kandemir et al. [32] reported that the Bax expression level in the hepatotoxicity induced by Cd significantly increased in the Cd-intoxicated group compared to other groups, while Bcl-2 expression decreased. The current findings are compatible with previous reports that Cd significantly increased the level of Bax expression and decreased Bcl-2 expression. Morin is known to have anti-apoptotic effects. A study determined that Morin normalized Bax and Bcl-2 levels in acrylamide-induced hepatotoxicity [32]. In our study, Morin prevented the increase in Bax expression and the decrease in Bcl-2 expression in a dose-dependent manner.

Consistent with our results, previous studies have shown that Cd causes severe histological changes in rat liver [60–62]. Here, histopathological data clearly showed that Morin significantly corrected the observed Cd-induced hepatic damage. Morin has been proven to have a protective effect on liver damage caused by different agents. Kandemir et al. [32] reported that Morin prevented tissue damage caused by acrylamide-induced hepatotoxicity in rats.

Mitogen-activated protein kinases (MAPKs) contain three primary members: c-Jun N-terminal kinase (JNK), extracellular signal-regulated kinase (ERK)1/2, and P38 MAPK. Cd causes the activation of JNK protein as a one of downstream signaling pathway that connects ER stress with apoptosis. This activation is also dependent on the cellular increase in ROS [63]. Wang et al. [64] showed that Cd increases ROS levels in the cell. This accelerates apoptosis by causing mitochondrial oxidative stress and an increase in JNK expression levels. Our study showed that the level of pro-apoptotic JNK expression increased in Cd-induced hepatotoxicity. Morin, by its anti-apoptotic properties in many tissues [47, 65], significantly reduced this increase.

## Conclusion

In conclusion, our findings demonstrated the hepatoprotective effect of morin against acute Cd intoxication with its antioxidant, anti-inflammatory, and anti-apoptotic actions. The study linked the underlying cellular mechanism to the modulation of upstream p-GRP78/PERK/ATF6 pro-apoptotic oxidative/ER stress and the downstream JNK/BAX/caspase 12 apoptotic signaling pathways. The results suggested the possible use of morin as a potential protective therapeutic candidate against oxidative stress, inflammation, and ER stress-mediated apoptosis following acute Cd-induced hepatic toxicity. The dose-dependent manner of Morin action requires further research to better understand the exact therapeutic dose.

## Data Availability

All the data and materials in the manuscript are available upon request.
